# Versican is upregulated in circulating monocytes in patients with systemic sclerosis and amplifies a CCL2-mediated pathogenic loop

**DOI:** 10.1186/ar4251

**Published:** 2013-07-11

**Authors:** Ayako Masuda, Hidekata Yasuoka, Takashi Satoh, Yuka Okazaki, Yukie Yamaguchi, Masataka Kuwana

**Affiliations:** 1 Department of Internal Medicine, Keio University School of Medicine, 35 Shinanomachi, Shunjuku, Tokyo 160-8582, Japan; 2 Yokohama City University School of Medicine, 3-9 Fukuura, Kanazawa-ku, Yokohama City, Kanagawa 236-0004, Japan

**Keywords:** systemic sclerosis, monocytes, versican, CCL2

## Abstract

**Introduction:**

Altered phenotypes of circulating monocytes of patients with systemic sclerosis (SSc) have been reported, but the role of these alterations in the pathogenesis of SSc remains unclear. This study was undertaken to identify molecules that are preferentially expressed by SSc monocytes, and to investigate the roles of these molecules in the pathogenic process of SSc.

**Methods:**

We analyzed circulating CD14^+ ^monocytes isolated from 36 patients with SSc and 32 healthy control subjects. The monocytes' gene expression profiles were assessed by Oligo GEArray^® ^(SABiosciences, Frederic, MA, USA) and semiquantitative or quantitative PCR; their protein expression was evaluated in culture supernatants of unstimulated monocytes by immunoblotting or ELISA, and by immunocytostaining. Monocyte chemoattractant activity of CCL2 was assessed in a TransWell^® ^system (Corning Incorporated, Corning, NY, USA) in the presence or absence of chondroitin sulfate (CS).

**Results:**

A step-wise approach to profiling gene expression identified that versican and CCL2 were upregulated in SSc monocytes. Subsequent analysis of proteins expressed in monocyte culture supernatants confirmed enhanced production of versican and CCL2 in SSc monocytes compared with control monocytes. CCL2 bound to CS chains of versican and colocalized with versican in the monocytes' Golgi apparatus. Finally, CCL2 had a greater ability to mediate monocyte migration when bound to CS chains, because this binding provided efficient formation of CCL2 gradients and protection from protease attack.

**Conclusion:**

Circulating monocytes with elevated versican and CCL2 levels may contribute to the fibrotic process in a subset of SSc patients by amplifying a positive feedback loop consisting of versican, CCL2, and the influx of monocytes.

## Introduction

Systemic sclerosis (SSc) is a multisystem disease characterized by microvascular abnormalities and excessive fibrosis [1]. Current research suggests that the pathogenic process of SSc damages endothelial cells and activates immune cells and fibroblasts, causing excessive accumulation of extracellular matrix (ECM) [2]. Mononuclear cell infiltration, consisting predominantly of macrophages and T cells, has been detected histopathologically in SSc lesions in the skin, lung, and other tissues, especially in the early phases of SSc [3,4]. Tissue macrophages in the perivascular skin express activation markers such as HLA-DR, platelet-derived growth factor B receptor [5], and CD163 [6]. Activated macrophages in the skin express CD204 [6], a marker for the M2 macrophages that are associated with wound repair and fibrotic conditions [7]. A recent study of lung tissue in SSc patients with interstitial lung disease found prominent infiltrates of fibrocytes expressing CD34, CD45, and collagen type I [8]; precursors of cells expressing these markers are found among circulating CD14^+ ^monocytes [9]. These findings indicate that monocytes and monocyte-lineage cells are actively involved in the pathophysiology of SSc.

Circulating CD14^+ ^monocytes derive from hematopoietic stem cells in the bone marrow and migrate to their ultimate sites of activity, and form a heterogeneous population in terms of surface markers, phagocytic capacity, and differentiation potential. Although circulating monocytes are committed precursors with the capacity to differentiate into a variety of phagocytes, including macrophages and dendritic cells, there is growing evidence that these monocytes can differentiate into other cell types as well, including cells with the typical characteristics of endothelial cells and fibroblasts [10-13]. Circulating monocytes are now recognized as multifunctional precursors, playing critical roles not only in immune and inflammatory responses but also in tissue regeneration and in pathologic tissue remodeling, such as excessive fibrosis and tumor development [13,14].

CD14^+ ^monocytes are increased in peripheral blood of SSc patients [6], and the molecular phenotypes and the proportions of cell types in the population are altered, with a larger proportion of type I collagen-producing monocytes [15], CXCR4^+ ^circulating cells with monocytic and endothelial features [16], monocytic proangiogenic hematopoietic cells [17], and CD163^+^CD204^+ ^cells with a profibrotic M2 phenotype [6]. Moreover, recent microarray analyses of circulating monocytes identified several genes that are overexpressed in SSc monocytes, including type I interferon-regulated genes such as Siglec-1 [18,19]. The SSc pathogenic process thus probably recruits circulating monocytes to the affected sites, where they acquire profibrotic properties. Although the details are still unclear, there may be at least two distinct mechanisms underlying profibrotic properties of these monocytes - the production of a variety of profibrotic growth factors, cytokines, and chemokines, including transforming growth factor beta and platelet-derived growth factor [2], and their transdifferentiation into ECM-producing cells [10,11,20].

In this study, we evaluated the gene and protein expression profiles of circulating CD14^+ ^monocytes in patients with SSc, using a high-throughput platform. We were particularly interested in genes related to ECM metabolism, chemokines, and their receptors, or endothelial cell function.

## Materials and methods

### Patients and controls

This study included 36 patients (four men and 32 women) who met the preliminary SSc classification criteria proposed by the American College of Rheumatology [21]. Using the published criteria, 19 patients were classified as having diffuse cutaneous SSc (dcSSc) and 17 as having limited cutaneous SSc (lcSSc) [22]. The study included 32 healthy control subjects (16 men and 16 women). The average age at the time of examination was 55.3 ± 15.9 in SSc patients and 45.8 ± 19.3 in control subjects.

Organ involvement related to SSc was defined for each patient as described previously [23]. SSc-related autoantibodies were identified by indirect immunofluorescence using commercially prepared slides of monolayer HEp-2 cells (MBL, Nagano, Japan) and immunoprecipitation assays [23]. The mean disease duration from onset of Raynaud's phenomenon was 13.6 ± 10.4 years. In patients with dcSSc, 13 of 19 were in late phase, with disease duration >5 years from the onset of non-Raynaud's phenomenon symptoms. Table 1 presents patients' autoantibody profiles, SSc-related organ involvement, and medications reported at the time of blood collection. Before collecting blood samples, we obtained written, informed consent from both patients and control subjects in accord with the tenets of the Declaration of Helsinki, and as approved by the International Review Board of Keio University.

**Table 1:** Clinical characteristics of 36 patients with systemic sclerosis

Characteristic	*n *(%)
Organ involvement	
Joint contractures	16 (44%)
Esophageal hypomotility	24 (67%)
Cardiac involvement	1 (3%)
Renal involvement	1 (3%)
Interstitial lung disease	22 (62%)
Pulmonary arterial hypertension	1 (3%)
Digital pitting scars	18 (50%)
Systemic sclerosis-related autoantibodies^a^	
Anti-topoisomerase I	16 (44%)
Anticentromere	7 (19%)
Anti-RNA polymerase III	2 (6%)
Anti-U1 ribonucleoprotein	7 (19%)
Anti-Th/To	2 (6%)
Not identified	5 (14%)
Medications reported when blood samples collected	
Prednisolone (≤10 mg/day)	11 (31%)
Cyclophosphamide	1 (3%)
Nonsteroidal anti-inflammatory drug	5 (14%)
D-Penicillamine	3 (8%)
Oral prostanoid	19 (53%)
Calcium channel blocker	2 (6%)
Statin	6 (17%)
Anti-platelet aggregation	6 (17%)
Antacid	12 (33%)

^a^Three patients had two systemic sclerosis-related autoantibodies: one patient with anti-topoisomerase I and anticentromere; one patient with anti-topoisomerase I and anti-U1 ribonucleoprotein; and one patient with anticentromere and anti-U1 ribonucleoprotein.

### Cell preparation

Peripheral blood mononuclear cells (PBMCs) were isolated from heparinized venous blood by Lymphoprep™ (Fresenius Kabi Norge, Halden, Norway) density-gradient centrifugation. CD14^+ ^monocytes were separated from PBMCs using an anti-CD14 mAb coupled to magnetic beads (CD14 MicroBeads; Miltenyi Biotech, Bergisch Gladbach, Germany) followed by magnetic cell sorting column separation according to the manufacturer's protocol [24]. Flow cytometric analysis showed that the sorted fraction consistently contained more than 95% CD14^+ ^cells.

### Gene expression profiling

Total RNA was extracted from purified monocytes using the ArrayGrade™ Total RNA Isolation kit (SABiosciences, Frederic, MA, USA) according to the manufacturer's protocol. Pooled RNA was prepared by mixing equal amounts of total RNA from five patients with SSc or from five healthy control subjects. We prepared two different independent sets of RNA. The first SSc patient set was composed of RNA from four females and one male with dcSSc (mean age at examination 40.0 ± 12.3, and mean disease duration from onset of Raynaud's phenomenon 9.4 ± 7.1 years). The second set was derived from four females and one male (four dcSSc and one lcSSc, mean age at examination 50.4 ± 8.9, and mean disease duration 16.2 ± 10.6 years). We generated biotin-16-uridine-5'-triphosphate-labeled cRNA probes from pooled total RNA (3 μg) using reverse transcription and a TrueLabeling-AMP™ 2.0 kit (SABiosciences). Gene expression was profiled from pooled total RNA (3 μg) using Oligo GEArray^® ^(SABiosciences) according to the manufacturer's instructions. This array covers 330 genes encoding ECM and adhesion molecules, chemokines and receptors, and proteins with endothelial cell functions.

The intensity of individual bands was measured by densitometry with National Institute of Health image software (Image J; National Institute of Mental Health, Bethesda, MD, USA). Relative gene expression levels were calculated as a ratio of the intensity of the target spot to that of glycelaldehyde-3-phosphate dehydrogenase. To identify genes that were upregulated in SSc monocytes, we compared the expression levels of individual genes in two independent sets of pooled monocyte RNA obtained from five SSc patients and from five healthy subjects. We selected candidate genes that met both of the following criteria: they were expressed at higher levels in SSc than in control monocytes in two independent sets, and they had 1.5-fold greater expression in SSc than in healthy monocytes in at least one set [25].

### Semiquantitative and quantitative PCR

Total RNA was extracted from monocytes using an RNeasy^® ^mini kit (Qiagen Inc, Valencia, CA, USA), first-strand cDNA was reverse-transcribed with an oligo (dT)_12-15 _primer (Invitrogen, Carlsbad, CA, USA), and cDNA equivalent to 2 ng total RNA was used for PCR analysis. The primer sequences, annealing temperatures, and cycles used to amplify individual genes are summarized in Table 2. Individual band intensity was quantified by densitometry. Relative mRNA expression levels were calculated as a ratio of the band intensity of the target gene to that of glyceraldehyde-3-phosphate dehydrogenase.

**Table 2:** Primer sequences, annealing temperatures, and cycles used for semiquantitative PCR

Gene	Sense primer (5' → 3')	Antisense primer (5' → 3')	Annealing temperature (ºC)	Cycle
*CCL2*	agcaagtgtcccaaagaagc	gcaatttccccaagtcctg	66	32
*Type I collagen α1*	cctggatgccatcaaagtct	ccttcttgaggttgccagtc	66	33
*Versican*	tcattcaacgtcaccttcca	ggtccaaaaatccaaaccaa	66	34
*L-selectin*	tcagctgctctgaaggaaca	taaccatgactgccactgga	60	30
*CCR1*	tcctcacgaaagcctacgaggagagtccaagc	ccacggagaggagggagccatttaac	66	30
*CXCL8*	cagttttgccaaggagtgct	attgcatctggcaaccctac	63	27
*MMP-2*	ccaaggagagctgcaacct	ccaaggtccatagctcatcgtc	63	40
*CCRL2*	ctgggctcatgctggggg	tgcagcagtgggtggtgg	60	30
*GAPDH*	tgaacgggaagctcactgg	tccaccaccctgttgctgta	60	25
Versican variants				
* Versican V0*	tcaacatctcatgttctccc	ttcttcactgtgggtataggtcta	57	34
* Versican V1*	ggctttgaccagtgcgattac	ttcttcactgtgggtataggtcta	57	28
* Versican V2*	tcaacatctcatgttctccc	ccagccatagtcacatgtctc	65	38
* Versican V3*	ggctttgaccagtgcgattac	ccagccatagtcacatgtctc	61	32

Gene mRNA expression levels were further evaluated by quantitative PCR using the TaqMan^® ^real-time PCR system (Applied Biosystems, Foster City, CA, USA) according to the manufacturer's protocols. Each gene's expression was measured relative to glyceraldehyde-3-phosphate dehydrogenase. Specific primers and probes for amplifying genes encoding L-selectin (Hs00174151), versican (Hs00171642), CCL2 (Hs00234140), CXCL8 (Hs00174103), versican V0 isoform (Hs01007944), and versican V1 isoform (Hs01007937) were purchased from Applied Biosystems. In some experiments, high and low mRNA expression levels were defined according to the mean plus two standard deviations of the levels in healthy control samples.

### Quantifying proteins in monocyte culture supernatants

Monocytes were plated and cultured in RPMI 1640 containing 10% fetal bovine serum, 50 U/ml penicillin, and 50 μg/ml streptomycin. To measure versican production, 5 × 10^6 ^monocytes were cultured in six-well plates without exogenous stimulation. Supernatants were harvested at 48 hours, concentrated with an Ultra-free MC 30K filter (Millipore, Billerica, MA, USA), and treated with chondroitinase ABC (Seikagaku Kogyo, Tokyo, Japan) to cleave chondroitin sulfate (CS) chains. The samples were then analyzed by SDS-PAGE, followed by immunoblotting with mouse mAb to human versican (clone 2B1; Seikagaku Kogyo) and horseradish peroxidase-conjugated goat anti-rabbit secondary antibodies (Thermo Fisher Scientific, Rockford, IL, USA). Bound antibodies were detected with a chemiluminescence detection system (Perkin Elmer Life Sciences, Boston, MA, USA). The signal intensity of the band corresponding to the molecular weight of a truncated versican (250 kDa) was quantified by densitometry. To measure CCL2 production, 10^5 ^cells were cultured in 24-well plates without exogenous stimulation, culture supernatants were harvested at 24 hours, and CCL2 was measured in culture supernatants using a Quantikine^® ^ELISA kit (R&D Systems, Abingdon, UK).

### Immunocytostaining

The intracellular localization of versican and CCL2 was determined by immunofluorescence as reported previously [26]. Briefly, CD14^+ ^monocytes were cultured on BD BioCoat™ Poly-D-Lysine Cellware (BD Biosciences, San Diego, CA, USA) for 2 hours. The cells were fixed with acetone and incubated with goat anti-human versican polyclonal antibodies (Santa Cruz Biotechnology, Santa Cruz, CA, USA) in combination with rabbit anti-human CCL2 polyclonal antibodies (Santa Cruz Biotechnology) or a mouse anti-human goldin-97 mAb (clone CDF4; Invitrogen), followed by incubation with the appropriate secondary antibodies conjugated to Alexa Fluor-488 or Alexa Fluor-568 (Invitrogen). For negative controls, cells were incubated with an isotype-matched mouse or rat mAb against an irrelevant antigen instead of the primary antibody. TO-PRO3 (Invitrogen) was used to counterstain nuclei. Images were taken with a Fluoview FV1000 confocal laser fluorescence microscope (Olympus, Tokyo, Japan).

### Assessing capacity of CCL2 for binding chondroitin sulfate

Carbonate buffer (15 mM Na_2_CO_3_, 10 mM NaHCO_3_) alone or a solution of synthetic CS (Seikagaku Kogyo) dissolved in carbonate buffer (200 μg/ml) was incubated in 24-well plastic plates overnight at 4°C. Unbound CS was removed, and recombinant CCL2 (50 ng/ml; R&D Systems) was added to the wells and incubated for 2 hours at 37°C. Protein components attached to the plate were recovered with 2% SDS, applied to immunoblots with rabbit anti-CCL2 polyclonal antibody (Abcam, Cambridge, MA, USA), and visualized with a chemiluminescence detection system.

To assess how binding to CS affected CCL2's vulnerability to protease-mediated degradation, we incubated recombinant CCL2 in 24-well plastic plates in the presence or absence of CS at 37°C for 2 hours, and then left the wells untreated or treated them with elastase (2 mM), cathepsin G (1 ml; Calbiochem, San Diego, CA, USA), or trypsin (0.0005%; BD Biosciences) at 37°C for 1 hour. Protein components were recovered and analyzed by immunoblots probed with anti-CCL2 polyclonal antibody. The signal intensity of the band corresponding to intact CCL2 (10 kDa) was semi-quantified using densitometry. The percentage of intact CCL2 in individual samples was expressed as a percentage of that found on pretreated CS-coated wells that did not receive protease treatment.

### Migration assay

Monocyte migration was evaluated as described previously [27], with some modifications. Briefly, the lower chambers of 24-well TransWell^® ^plates with 5 μm pore filters (Corning Incorporated, Corning, NY, USA) were left untreated (vehicle) or coated with serial concentrations of CS (10, 50, and 250 μg/ml). The wells were incubated with recombinant CCL2 (50 ng/ml) for 2 hours at 37°C, after which monocytes (3 × 10^5^) were placed in the upper chambers for 2 hours at 37°C with 5% carbon dioxide. Cells in the lower chambers were counted manually using a hemocytometer, and migration ratios were calculated as a percentage of the cells induced to migrate by vehicle alone. All experiments were carried out in duplicate. In some experiments, mouse anti-CCL2 mAb (R&D Systems) or mouse IgG (3.0 ng/ml; Dako, Glostrup, Demark) was added to the lower chamber. The relative monocyte migration in individual experiments was calculated as a percentage of the migration induced in vehicle-coated wells without CCL2.

### Statistical analysis

All continuous variables were recorded as mean ± standard deviation, and statistical differences were compared using a nonparametric Mann-Whitney U test. Categorical variables were compared with Fisher's exact test or a chi-square test when appropriate. The correlation coefficient (*r*) was determined using a single regression model.

## Results

### Identifying genes with altered expression in SSc monocytes

**Figure 1:**
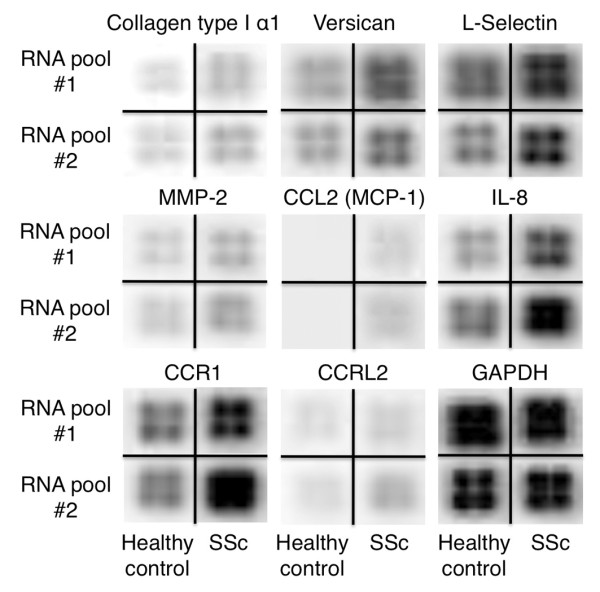
**Gene expression profiles of circulating monocytes from patients with systemic sclerosis and from healthy controls**. Two sets of genes, each set containing RNA from five subjects, were analyzed (RNA pool #1 and #2) with the Oligo GEArray^® ^(SABiosciences, Frederic, MA, USA). The results for eight candidate genes for genes preferentially overexpressed in systemic sclerosis (SSc) monocytes are shown along with control (glycelaldehyde-3-phosphate dehydrogenase (GAPDH)).

We used the Oligo GEarray™ system, which can screen 330 genes associated with ECM and adhesion molecules, chemokines and receptors, and endothelial cell biology, to compare gene expression profiles in circulating monocytes from SSc patients or healthy controls. We performed two independent analysis sets on mixed total RNA samples: one set from five SSc patients and the other from five healthy controls. Based on results of two independent sets of analysis, we selected collagen type I α1, versican, L-selectin, matrix metalloproteinase-2, CCL2, CXCL8, CCR1, and CCRL2 as candidates for genes preferentially overexpressed in SSc monocytes (Figure 1). Of these, only versican was confirmed by semiquantitative PCR and quantitative TaqMan^® ^real-time PCR to be significantly upregulated in SSc monocytes.

**Figure 2:**
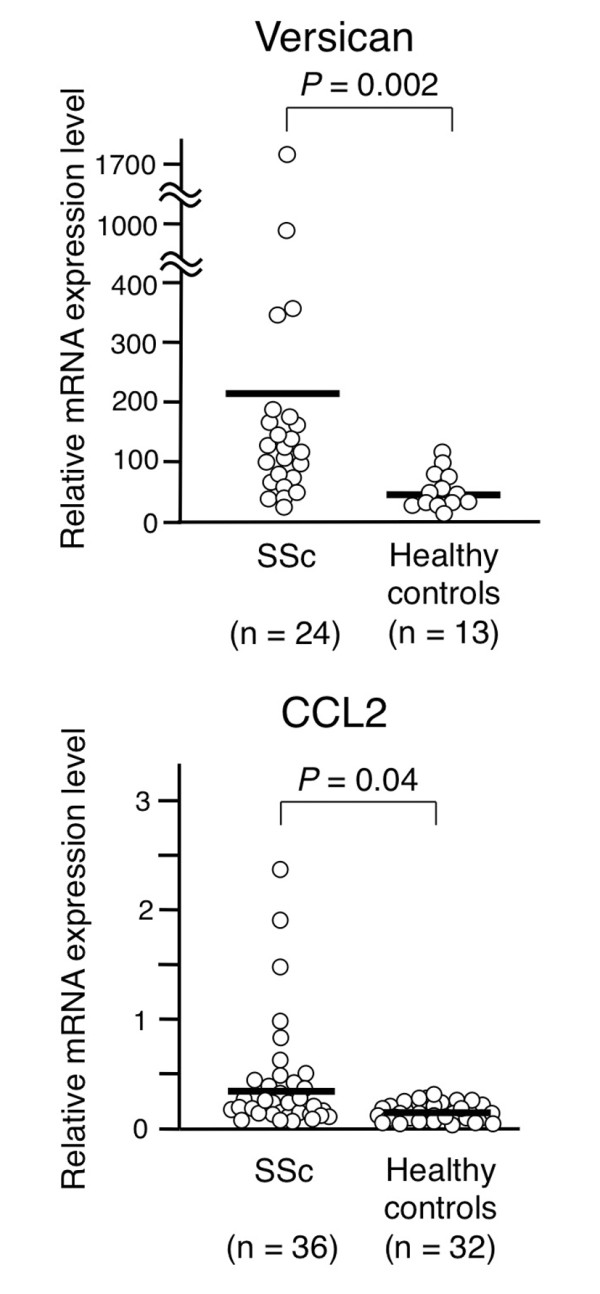
**Versican and CCL2 mRNA levels in monocytes from patients with systemic sclerosis and healthy controls**. Quantitative PCR analysis. Relative mRNA expression levels were calculated as a ratio of mRNA levels of the genes of interest to those of glyceraldehyde-3-phosphate dehydrogenase (GAPDH). Bars in the graph denote the mean. Differences between the groups were analyzed by Mann-Whitney *U *test. SSc, systemic sclerosis.

Figure 2 shows versican mRNA levels, quantified by TaqMan^® ^real-time PCR, in monocytes from 24 SSc patients and 13 control subjects (219.9 ± 376.5 vs. 46.2 ± 31.1, *P *= 0.002). Although CCL2 expression tended to be higher in SSc patients, the difference was not statistically significant (*P *= 0.06). Since CCL2 levels in SSc monocytes varied widely, we increased the number of subjects sampled (36 patients with SSc, 32 control subjects) and the difference in CCL2 gene expression between the two groups reached statistical significance (0.37 ± 0.53 vs. 0.11 ± 0.07, *P *= 0.04) (Figure 2). The remaining six candidate genes were excluded because confirmatory analyses did not show a statistically significant difference between their expression levels in SSc and control monocytes.

### Clinical features associated with high versican or CCL2 mRNA expression in monocytes

Versican and CCL2 mRNA levels varied considerably among SSc monocytes, and high expression levels were detected in a subgroup of patients. We examined clinical features associated with a high level of versican or CCL2 mRNA in circulating monocytes, which were defined based on above the mean plus two standard deviations of the levels in healthy control samples. We examined 24 patients with SSc, 11 with high levels and 13 with low levels of versican expression, and found differences in the frequencies of dcSSc (82% vs. 25%, *P *= 0.02), interstitial lung disease (82% vs. 46%, *P *= 0.04), positive anti-topoisomerase I antibody (64% vs. 15%, *P *= 0.01), and esophageal involvement (100% vs. 46%, *P *= 0.006). In fact, versican levels were significantly higher in patients with dcSSc than in those with lcSSc (413 ± 531 vs. 100 ± 92, *P *= 0.03), and in patients with esophageal involvement than those without (340 ± 459 vs. 54 ± 34, *P *= 0.002). In particular, all four patients with an extremely high mRNA expression level of versican (>300) had dcSSc. We did not find any correlation with clinical characteristics and the level of CCL2 mRNA expressed by circulating monocytes in SSc patients.

### Upregulated mRNA expression of the versican isoforms V0 and V1 in SSc monocytes

**Figure 3:**
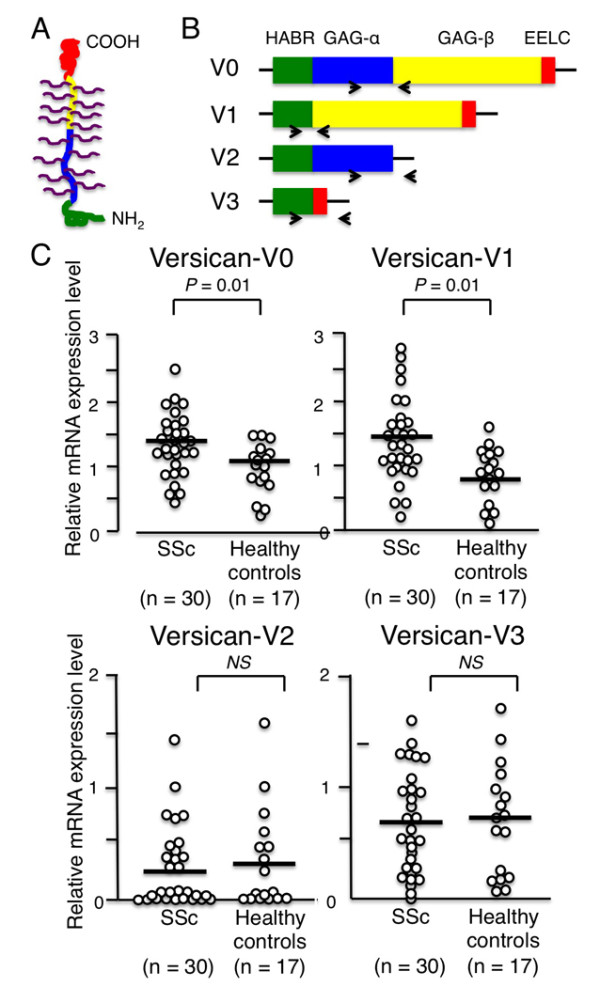
**Versican splice variants and mRNA levels in monocytes from systemic sclerosis patients and healthy controls**. **(A) **Molecular structure of full-length versican (V0), which has numerous chondroitin sulfate (CS) chains attached to its glycosaminoglycan (GAG)-binding domain. **(B) **mRNA structures of versican splice variants (V0, V1, V2 and V3). Versican is composed of a hyaluronan binding region (HABR, green), GAG-binding domains (blue and yellow), and epidermal growth factor-like, lectin-like, and complement-regulatory-like domains (EELC, red). Individual mRNA components are shown in the same color as their corresponding protein structures. Arrows denote primers used to amplify each splice variant. **(C) **Levels of versican V0, V1, V2, and V3 mRNA in systemic sclerosis (SSc) and control monocytes, analyzed using semi-quantitative PCR. Relative mRNA levels were calculated as a ratio of the expression level of the gene of interest to that of glyceraldehyde-3-phosphate dehydrogenase (GAPDH). Each bar in the graph denotes the mean. Differences between the two groups were analyzed by Mann-Whitney *U *test. NS, not significant.

Versican, or CS proteoglycan 2, is a large extracellular matrix proteoglycan (>1,000 kDa) that is present in a variety of human tissues, including skin and blood vessels [28]. Versican consists of an amino-terminal hyaluronan binding region, a glycosaminoglycan (GAG)-binding domain, and a C-type lectin-like domain. Numerous CS chains are attached to a GAG-binding domain (Figure 3A). In addition to full-length versican (V0), three short isoforms having GAG-binding domains of different sizes (V1, V2, and V3) are generated by alternative splicing (Figure 3B).

We designed PCR primers to detect each of the four versican isoforms separately, and assessed their mRNA levels in SSc and control monocytes. Semiquantitative PCR analysis of monocytes from 30 patients with SSc and 17 healthy controls showed significantly higher mRNA levels of both V0 and V1 in SSc than in control monocytes (*P *= 0.01 for both comparisons), while V2 and V3 levels were comparable in the two groups (Figure 3C).

**Figure 4:**
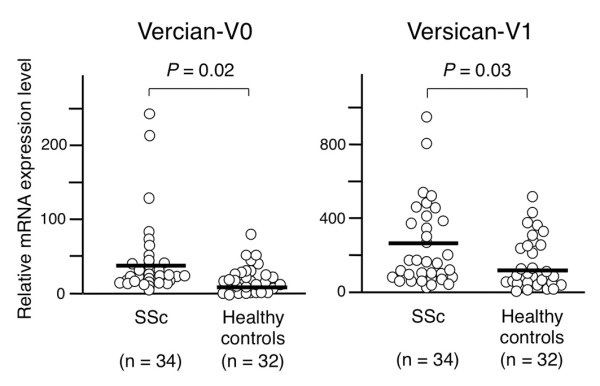
**Versican V0 and V1 mRNA levels in systemic sclerosis and control monocytes**. Quantitative PCR analysis. Relative mRNA levels were calculated as a ratio of the level of the gene of interest to that of glyceraldehyde-3-phosphate dehydrogenase (GAPDH). Each bar in the graph denotes the mean. Differences between the groups were analyzed by Mann-Whitney *U *test. SSc, systemic sclerosis.

TaqMan^® ^real-time PCR confirmed that mRNA expression of the V0 and V1 isoforms, both of which have long GAG-binding domains, was upregulated in SSc patients as compared with healthy control subjects (Figure 4).

### Versican and CCL2 proteins are upregulated in SSc monocytes

**Figure 5:**
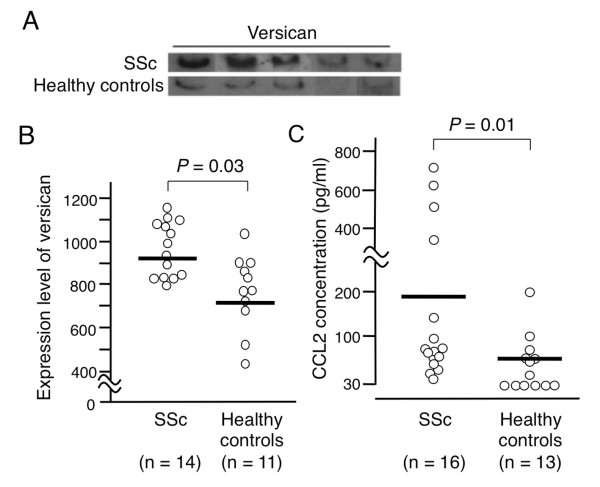
**Versican V0 and CCL2 protein levels in monocyte culture supernatants**. Versican V0 and CCL2 protein levels in monocyte culture supernatants derived from systemic sclerosis (SSc) patients and healthy controls. **(A) **Representative immunoblot evaluating versican V0 protein expression. Monocyte culture supernatants were concentrated, truncated, and applied to immunoblots. **(B) **Versican V0 protein levels in monocytes from 14 patients with SSc and 11 healthy controls, semiquantitatively measured by densitometry. **(C) **CCL2 protein levels in monocytes from 16 patients with SSc and 13 healthy controls. CCL concentration in culture supernatants was measured by an ELISA. Each bar in the graph denotes the mean. Results from the two groups were compared by Mann-Whitney *U *test.

We cultured freshly isolated monocytes without any exogenous stimuli, and measured versican and CCL2 proteins spontaneously secreted into the supernatant during cultures. As shown in Figure 5A, the versican V0 isoform was concentrated and detected in supernatants by immunoblotting. Versican levels were significantly higher in culture supernatants from SSc monocytes than in those from healthy control monocytes (*P *= 0.03) (Figure 5B). SSc monocytes also produced more CCL2 than did control monocytes (*P *= 0.01) (Figure 5C). The mRNA and protein expression levels in a given patient were correlated with each other for versican (*r*^2 ^= 0.66, *P *= 0.003) and CCL2 (*r*^2 ^= 0.51, *P *= 0.004).

### Capacity of CCL2 to bind chondroitin sulfate chains

**Figure 6:**
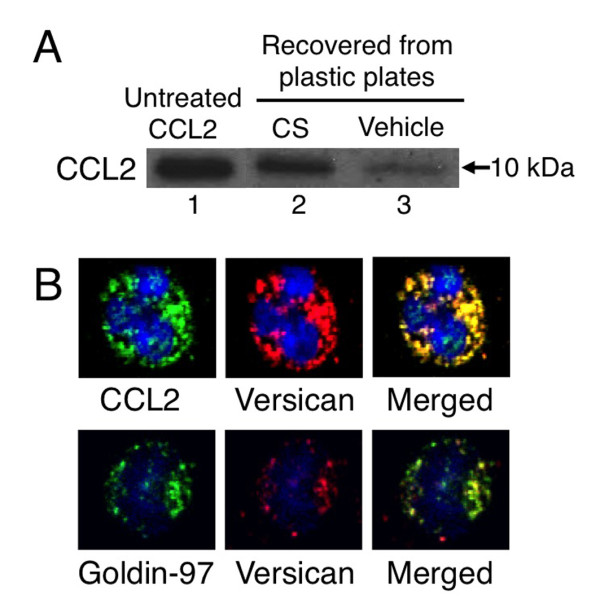
**Formation of CCL2 and versican complex in circulating monocytes**. **(A) **CCL2 was incubated with plastic plates coated with chondroitin sulfate (CS) or vehicle. Bound CCL2 was recovered and subjected to immunoblots (lanes 2 and 3, respectively). Lane 1, untreated CCL2 as a positive control. A representative result from three experiments is shown. **(B) **Versican and CCL2 cellular localization in monocytes from an systemic sclerosis (SSc) patient, assessed by multi-color immunocytostaining: upper panel, CCL2 (green), versican (red), and their merged image; lower panel, goldin-97 (green), versican (red), and their merged image. Nuclei were counterstained with TO-PRO3 (blue). A representative result from three independent experiments is shown. Original magnification, ×600.

Versican's negatively charged CS chains can bind to chemokines such as CCL2, CCL3, and CCL5 via ionic interactions, and can function as a chemokine reservoir [28,29]. The versican isoforms V0 and V1, which were both elevated in SSc monocytes, have numerous CS chains attached to the GAG-binding domain and thus have a large capacity for binding chemokines [28]. Both versican and CCL2 are upregulated in SSc monocytes; to determine whether these form a complex, we examined CCL2's binding capacity using plastic plates coated with or without synthetic CS (Figure 6A). As expected, CCL2 was able to bind to the plates only when CS was present. Immunocytostaining showed the cellular localization of versican and CCL2 in the Golgi apparatus of monocytes; the representative images of SSc monocytes in Figure 6B show versican and CCL2 colocalized in the Golgi apparatus. Additional experiments using monocytes derived from a patient with SSc and a healthy control subject produced concordant findings. Since the Golgi plays an important role in the synthesis of proteoglycans [30], these results suggest that versican forms a complex with CCL2 before secretion by monocytes.

### Chondroitin sulfate chains enhance CCL2-mediated monocyte migration

**Figure 7:**
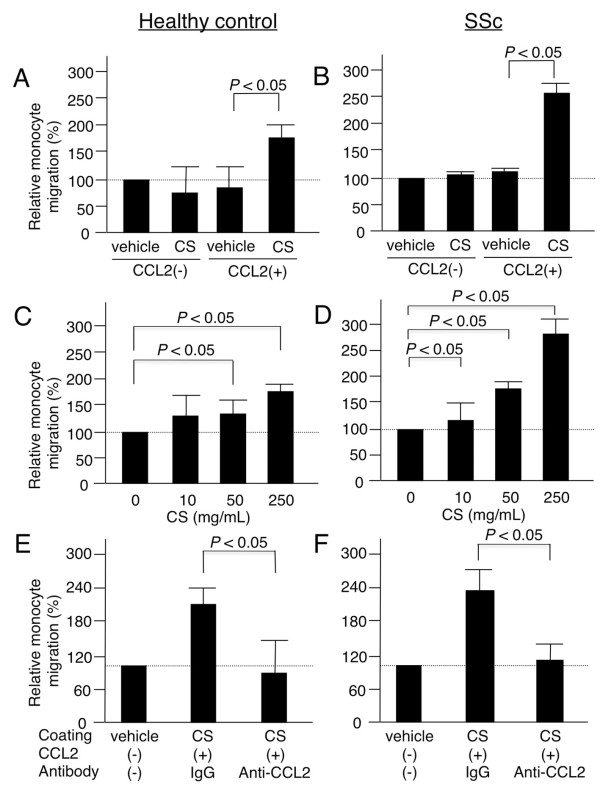
**CCL2 chemoattractant activity promoting monocyte migration, with or without chondroitin sulfate binding**. Circulating monocytes derived from healthy controls (A, C, E) and systemic sclerosis (SSc) patients (B, D, F). **(A, B) **CCL2-induced monocyte migration in the presence or absence of chondroitin sulfate (CS) coating. Lower chambers of a TransWell^® ^double-chamber system (Corning Incorporated, Corning, NY, USA) were coated with CS or vehicle, and CD14^+ ^monocytes were applied to the upper chambers. **(C, D) **CCL2-induced monocyte migration on plastic plates precoated with serial concentrations of CS. **(E, F) **CCL2-induced monocyte migration on CS-coated plastic plates in the presence of anti-CCL2 mAb or control IgG. Relative monocyte migration was calculated as a percentage of migration in a control experiment using vehicle-coated wells without CCL2. All experiments were carried out in duplicate; the mean and standard deviation of three measurements is shown. Results from the two groups were compared using a Mann-Whitney *U *test. A representative result from four independent experiments is shown.

The chemokine CCL2 induces monocytes, neutrophils, and lymphocytes to migrate [31]. To examine whether CCL2's capacity to induce migration is enhanced by binding to versican's CS chains, we performed migration assays in a TransWell^® ^double-chamber system using CD14^+ ^monocytes derived from healthy controls and SSc patients. First, monocytes were cultured in the upper chamber, and the lower chamber was pre-coated with CS or vehicle alone in the presence or absence of CCL2 (Figure 7A,B). Monocyte migration was promoted only in the presence of CS-coated plates treated with CCL2, and the strength of the effect depended on the CS concentration in the coating (Figure 7C,D). This enhanced monocyte migration was completely blocked by adding an anti-CCL2 neutralizing antibody (Figure 7E,F). Monocytes derived from a healthy control and an SSc patient showed the similar behavior. Concordant results were obtained in additional experiments using monocytes derived from three healthy controls and three SSc patients. These findings indicate that CCL2 activity is augmented by binding to CS chains.

**Figure 8:**
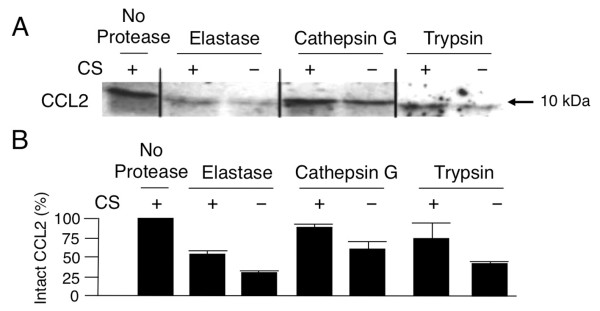
**Binding to chondroitin sulfate chains protects CCL2 from protease-mediated degradation**. **(A) **CCL2 was pre-incubated with chondroitin sulfate (CS) or vehicle, and treated with a series of proteases including elastase, cathepsin G, and trypsin. A representative immunoblot from three different experiments shows a band corresponding to intact CCL2 (10 kDa). **(B) **Amount of intact CCL2 in individual wells precoated with CS or vehicle, and subsequently treated with a series of proteases. Mean and standard deviation of three independent measurements is shown. The quantity of intact CCL2 in individual samples was expressed as a percentage of the quantity of CCL2 on wells precoated with CS and not treated with protease.

Enhanced CCL2-mediated monocyte migration was probably due to the efficient formation of a chemotactic gradient, but it was also possible that CCL2 was protected from degradation when bound to a CS chain. To test this hypothesis, CCL2 was incubated with plates treated with CS or vehicle, and exposed to a variety of proteases that included elastase, cathepsin G, and trypsin. As shown in Figure 8A, CCL2 was degraded in the presence of proteases, but was protected from protease-mediated degradation when bound to the CS-coated plates. Concordant findings were obtained from three healthy controls (Figure 8B).

## Discussion

This study has demonstrated that versican and CCL2 are upregulated in circulating CD14^+ ^monocytes in a subset of SSc patients. High versican levels in circulating monocytes were associated with fibrotic characteristics of SSc, such as diffuse cutaneous involvement. Interestingly, versican forms a reservoir for various CC chemokines that induce the migration of circulating monocytes, which produce additional versican after arriving at the versican-rich site. CCL2's ability to induce monocytes to migrate is enhanced by its binding to versican, due to the efficient formation of chemokine gradients and protection from proteolytic degradation. This positive feedback loop, consisting of versican, CCL2, and the influx of monocytes, may be enhanced at the affected sites of a subset of SSc patients with phenotypically altered circulating monocytes.

Versican is involved in many physiologic and pathologic processes, including neuronal development [32], atherosclerosis [33], and the invasive and metastatic signatures of many cancers [34]. As with other ECM components, versican is enriched in the skin of patients with SSc [35], although little is known about versican's role in SSc pathogenesis. Versican is able to bind type I collagen and hyaluronic acid to maintain the integrity of the ECM [28], which may be important in forming the stiff fibrotic tissue seen with SSc. Versican also functions as a unique reservoir for a variety of growth factors, chemokines, and cytokines, which it gathers via numerous CS chains attached to its GAG-binding domain [28]. Chemokines known to bind versican include CCL2, CCL3, CCL5, CCL21, CXCL10, and CXCL12. It is particularly noteworthy that versican generates a chemotactic gradient of CC chemokines that attracts circulating monocytes and T cells to versican-rich sites [36]. Versican subsequently promotes the adhesion and activation of recruited monocytes through binding to adhesion receptors such as integrins and CD44 on cell surfaces [37]. Through these processes, versican generates a complex set of environmental cues for infiltrating mononuclear cells and resident cells [28].

Interestingly, monocytes and monocyte-derived cells are not only recruited by versican, but are also major producers of versican [38,39]. Therefore, after migrating to versican-rich sites, these monocytes amplify the tissue response by producing more versican, which in turn promotes the influx of more monocytes [40]. In this regard, recent studies indicate that the positive feedback loop formed by versican and monocyte-lineage cells is critical for inducing certain pathologic conditions, such as tumor invasion and metastasis [41] and the formation of atherosclerotic plaques [38]. In patients with SSc, this versican-mediated positive feedback loop probably contributes to the fibrotic process by recruiting certain subsets of monocytes that acquire profibrotic properties [6,15]. The mechanisms that initially stimulate the release of versican in the early stages of SSc remain elusive, but once this positive feedback loop is established the profibrotic response would, theoretically, be amplified endlessly.

CCL2, a chemokine known to be involved in pathogenic process of SSc [42], is also elevated in circulating monocytes from SSc patients. Levels of circulating CCL2 were elevated in SSc patients, especially in those with early dcSSc [43,44] or interstitial lung disease [43,45,46]. A recent longitudinal analysis in patients with dcSSc found that circulating CCL2 decreases year after year, along with improvements in skin sclerosis [47], suggesting CCL2 as an indicator of profibrotic activity in patients with SSc. Several animal models of tissue fibrosis have demonstrated CCL2's crucial role in the fibrotic process, in which attenuating CCL2 activity prevents tissue fibrosis. Mice lacking CCL2 are protected from bleomycin-induced dermal fibrosis [48], while mice lacking the CCL2 receptor CCR2 are protected from bleomycin-induced lung fibrosis [49,50]. In these models, monocyte infiltration and collagen deposition were remarkably lower than in wild-type mice. The present study raises the question of which cell type producing CCL2 is more likely to be important for SSc pathogenesis. In this regard, abundant expression of CCL2 was observed in fibroblasts and mononuclear cells in the skin of SSc patients [45,51,52]. CCL2 has also been reported to be expressed mainly by infiltrating monocytes early in the disease, whereas fibroblasts become the major source for CCL2 in the skin later in the disease [42]. Unfortunately, our *ex vivo *analysis failed to demonstrate which cell type is the primary source of CCL2 involved in the pathogenic process of SSc.

Despite this definitive role of CCL2 in the development of excessive fibrosis *in vivo*, the details of the profibrotic mechanisms remain unclear. Yamamoto and colleagues reported that CCL2 significantly increased the levels of collagen mRNA expressed in cultured dermal fibroblasts [53], but another study failed to reproduce this finding [54]. While CCR2 is generally not expressed by fibroblasts, Carulli and colleagues found that CCR2 is expressed by a small population of fibroblasts derived from patients with early dcSSc [55]. CCL2's profibrotic effects may thus require interaction with other cell types that express CCR2, such as monocytes and T cells. In this regard, it has been shown in SSc patients that CCL2 induces infiltrating CD4^+ ^T cells to differentiate into T-helper 2 cells, which release higher amounts of IL-4 and stimulate fibroblasts to produce excess ECM [54]. In this scenario, versican functions as a local reservoir for CCL2 and, whether bound to or released from versican, CCL2 is capable of efficiently stimulating T cells. In addition to its role in this T-cell-mediated mechanism, CCL2 also contributes to fibrotic response by promoting the migration and accumulation of profibrotic monocytes at affected sites in SSc patients. All together, the upregulation of versican and CCL2 in circulating monocytes accelerates CCL2-mediated profibrotic responses in SSc patients.

What mechanisms assist in shaping circulating monocytes to the profibrotic phenotype seen in patients with SSc? One of histopathological hallmarks of SSc is the perivascular infiltration of monocytes early in the disease [4]. It is possible that intrinsically altered monocytes migrate into target organs and trigger a profibrotic response by stimulating resident fibroblasts. Alternatively, monocyte phenotypes may be altered in SSc patients by the strong profibrotic environment. Versican production is highly regulated by soluble factors and certain stimuli, and several studies have reported that profibrotic growth factors such as transforming growth factor beta, platelet-derived growth factor, and basic fibroblast growth factor upregulate versican synthesis [56-58]. These factors are also known to upregulate CCL2 expression [59,60]. In contrast, IFNγ and IL-1β reduce versican expression [61,62]. Hypoxia dramatically upregulates versican in macrophages via hypoxia-inducible factor signaling [38]. The profibrotic and hypoxic environment associated with SSc may modulate gene expression profiles of circulating monocytes.

Genes selected by initial screening via gene expression array but excluded by confirmatory analyses may be of some interest, because some of them have been reported as molecules associated with SSc pathogenesis. For example, circulating levels of soluble L-selectin and CXCL8 were increased in SSc patients versus healthy controls [63,64]. In addition, gene expression of CCR1 was shown to be upregulated in PBMCs derived from patients with lcSSc and pulmonary arterial hypertension [65].

There are several limitations to this study. First, we used total CD14^+ ^monocytes enriched form PBMCs, which contained CD14^+ ^fibrocyte precursors [9]. Since fibrocyte precursors are reported to be a rare cell population, comprising approximately 0.5% of circulating monocytes [66], contamination of them into CD14^+ ^cells should have minimal impact on gene and protein expression data. Second, prominent upregulation of versican and CCL2 in circulating monocytes was observed in a minority of SSc patients, raising a possibility that the monocytic versican-mediated pathogenic process is only one of the roles of circulating monocytes in the pathogenesis of SSc. Since all patients with an extremely high mRNA expression level of versican had dcSSc, this type of monocyte phenotypic change might be unique to patients with a prominent fibrotic phenotype. Finally, this study represented only an *in vitro *functional interaction between versican and CCL2, which may not reflect *in vivo *activity. In addition, overexpression of versican in circulating monocytes of SSc patients might be a bystander of other more important pathogenic process of SSc. Further investigations involving genetically manipulated animals- for example, mice lacking functional versican expression specifically in monocytes - is necessary to confirm a critical role of versican upregulated by circulating monocytes in SSc pathogenesis.

## Conclusion

The cellular and molecular mechanisms underlying the SSc fibrotic process primarily involve the interaction of cells such as fibroblasts, endothelial cells, and circulating immune cells, orchestrated by profibrotic soluble mediators and ECM components. This concept is supported by our observation that circulating monocytes in SSc patients are phenotypically altered and amplify a positive feedback loop, mediated by versican and CCL2, between monocytes and fibroblasts. Further studies evaluating the roles of circulating monocytes in the pathogenic process of SSc should help to elucidate the complex pathophysiology of SSc and assist us to develop novel therapeutic strategies in this multisystem fibrotic disease.

## Abbreviations

CS: chondroitin sulfate; dcSSC: diffuse cutaneous systemic sclerosis; ECM: extracellular matrix: ELISA: enzyme-linked immunosorbent assay; GAG: glycosaminoglycan; IFN: interferon; IL: interleukin; lcSSc: limited cutaneous systemic sclerosis; mAb: monoclonal antibody; PBMC: peripheral blood mononuclear cell; PCR: polymerase chain reaction; RT: reverse transcriptase; SSc: systemic sclerosis.

## Competing interests

The authors declare that they have no competing interests.

## Authors' contributions

AM acquired: analyzed, and interpreted data, and wrote the manuscript. HY analyzed and interpreted data, and wrote the manuscript. TS, YO, and YY acquired data. MK designed the experiments, analyzed and interpreted data, and wrote the manuscript. All authors read and approved the final manuscript.
